# Evaluation of the expression of necroptosis pathway mediators and its association with tumor characteristics in functional and non-functional pituitary adenomas

**DOI:** 10.1186/s12902-021-00919-y

**Published:** 2022-01-04

**Authors:** Mohammad E. Khamseh, Alireza Sheikhi, Zahra Shahsavari, Mohammad Ghorbani, Hamideh Akbari, Mehrnaz Imani, Mahshid Panahi, Alimohammad Alimohammadi, Maryam Ameri, Shima Nazem, Vahid Salimi, Masoumeh Tavakoli-Yaraki

**Affiliations:** 1 Endocrine Research Center, Institute of Endocrinology and Metabolism, Iran University of Medical Sciences, Tehran, Iran; 2 Department of Biochemistry, School of Medicine, Iran University of Medical Sciences, Tehran, Iran; 3 Department of Clinical Biochemistry, Faculty of Medicine, Shahid Beheshti University of Medical Sciences, Tehran, Iran; 4 Division of Vascular and Endovascular Neurosurgery, Firoozgar Hospital, Iran University of Medical Sciences, Tehran, Iran; 5 Clinical Research Development Unit (CRDU), Sayad Shirazi Hospital, Golestan University of Medical Sciences, Gorgan, Iran; 6 Firozgar Hospital, Pathology Department, Iran University of Medical Sciences, Tehran, Iran; 7 Iranian Legal Medicine Organizations, Tehran, Iran; 8 Forensic Medicine Department, Faculty of Medicine, Iran University of Medical Sciences, Tehran, Iran; 9 Department of Laboratory Medicine, Faculty of Paramedical Sciences, Shahid Beheshti University of Medical Sciences, Tehran, Iran; 10 Department of Virology, School of Public Health, Tehran University of Medical Sciences, Tehran, Iran

**Keywords:** Pituitary adenoma, Functional pituitary adenomas, Non-functional pituitary adenomas, Necroptosis

## Abstract

**Background:**

Pituitary adenomas impose a burden of morbidity on patients and characterizing the molecular mechanisms underlying its pathogenesis received remarkable attention. Despite the appealing role of necroptosis as an alternative cell death pathway in cancer pathogenesis, its relevance to pituitary adenoma pathogenesis has yet to be determined that is perused in the current study.

**Methods:**

The total number of 109 specimens including pituitary adenomas and cadaveric healthy pituitary tissues were enrolled in the current study. Tumor and healthy pituitary tissues were subjected to RNA extraction and gene analysis using Real-Time PCR. The expression levels of necroptosis markers (RIP1K, RIP3K and, MLKL) and their association with the patient’s demographic features were evaluated, also the protein level of MLKL was assessed using immunohistochemistry in tissues.

**Results:**

Based on our data, the remarkable reduction in RIP3K and MLKL expression were detected in nonfunctional and GH-secreting pituitary tumors compared to pituitary normal tissues. Invasive tumors revealed lower expression of RIP3K and MLKL compared to non-invasive tumors, also the attenuated level of MLKL was associated with the tumor size in invasive NFPA. The simultaneous down-regulation of MLKL protein in pituitary adenoma tissues was observed which was in line with its gene expression. While, RIP1K over-expressed significantly in both types of pituitary tumors which showed no significant correlation with patient’s age, gender and tumor size in GHPPA and NFPA group. Notably, MLKL and RIP3K gene expression was significantly correlated in the GHPPA group.

**Conclusions:**

According to our data, the reduced expression of necroptosis mediators (RIP3K, MLKL) in pituitary adenoma reinforces the hypothesis that the necroptosis pathway can be effective in regulating the proliferation and growth of pituitary tumor cells and tumor recurrence.

## Background

Pituitary adenomas are the slow-growing, relatively common and almost benign tumors which account for up to 16% of the intracranial neoplasms worldwide [1]. The uncontrolled proliferation of anterior pituitary gland cells imposes the heavy burden of morbidity on patients due to the remarkable role of pituitary as a master gland in human health and metabolism [2]. The excessive hormone secretion, compression of optic chiasm, vision loss and headache are the most common symptoms of pituitary adenomas [3–5]. Among all treatment protocols, surgical resection is the most preferred and effective therapeutic approaches which can accompany with tumor recurrence in almost one third of cases [6]. Tumor cell owes its fate to the loss of death and gain of proliferation which provide appropriate cell growth condition and progression [7]. Distinct cell death pathways have been recognized including apoptosis, autophagy and necroptosis [8–10]. More recently, necroptosis, known as a programmed necrosis has attained considerable interests in the regulation of cancer cell growth with different biochemical and morphological features comparing to other cell death pathways [11–13]. Receptor-interacting protein kinases (RIPKs) are the key regulatory serine/threonine kinases which regulate and mediate necroptosis pathway. Triggering of tumor necrosis factor (TNF) receptor and adaptor proteins likewise TNFRSF1A-associated death domain (TRADD), Fas-associated death domain (FADD) lead to the interaction of RIP1K and RIP3K through respective homotypic interaction motif (RHIM domain). Interaction of RIP1K and RIP3K results in phosphorylation and activation of RIP3K and recruitment of mixed lineage kinase domain-like (MLKL) which is the other hallmark of necroptosis pathway. Phosphorylation of MLKL by RIP3K induces structural changes in the plasma membrane and further ionic channels formation leading to the membrane disruption and necroptosis execution [14, 15]. Increased resistance to apoptosis, as the main way of cancer cell death open up an insight into trigger alternative death-inducing approaches for controlling tumor cell growth. In support of this, it has been shown that the expression level of RIP3K was lower in malignant breast cancer tumors which were correlated with tumor grade [16]. Moreover, activation of necroptosis pathway via shikonin was associated with elevated intracellular ROS level, higher RIP1K and RIP3K expressions and reduced mitochondrial membrane potential in the triple negative breast cancer cell line [17]. Similarly, overproduction of ROS following shikonin-induced necroptosis in glioma cells has emphasized on the regulatory and executor role of ROS in the RIP1K-RIP3K mediated necroptosis [18]. The anti-tumor effect of shikonin was elucidated in primary and metastatic bone tumors via elevation of RIP1K- RIP3K protein levels and no activation of apoptosis markers likewise caspase 3 and 6 [19]. The involvement of apoptosis in development and cell transformation in favor of tumor formation in pituitary gland has been surveyed in few studies [20]. Notably, Kontogeorgos has reported the higher rate of apoptosis in functional, microadenoma, aggressive and drug-resistance pituitary adenoma [21]. Additionally, it has been shown that the expression of Bcl-2 protein (an anti-apoptotic element) was decreased in prolactinoma and NFPA while elevated in hormone-secreting adenomas also the level of Bax protein (a pro-apoptotic element) was reduced in recurrent tumors [22]. The study of Sambaziotis et al*,* has revealed the higher expression of Bcl-2 protein in NFPA as well as higher level of Bax protein in FPA comparing to the rest of pituitary adenomas [23]. Contradictions exist regarding the exact role of death pathways in the progression of pituitary adenomas which require further elucidations. The un-known relevance of necroptosis, as a recent appealing rout of cell death beside the essence of characterizing molecular mechanisms driving pituitary tumor formation provoked us to delineate the expression pattern of necroptotic cell death biomarkers in the most prevalent pituitary adenomas and their association with patient’s clinic pathological properties.

## Methods

### Patients and sample collection

The total number of 109 specimens including, 79 pituitary adenomas and 30 cadaveric healthy pituitary tissues were included in this study with local ethical approval and informed consent. Following the ethical standards in Declaration of Helsinki, the project was ethically approved by the ethics committee of Vice president of research of Iran University of Medical Sciences. All specimens of pituitary adenomas obtained from patients whom were diagnosed for pituitary adenoma and subjected to the endoscopic transnasal transphenoidal surgery (ETSS) at the neurosurgery department of our institute from January 2017 to June 2020 and patients with a known history of any malignancy were excluded from the study. Notably, patients who received no treatments before surgery were included in this study to avoid any possible effect of medication on necroptosis mediators. The imaging, post-surgery pathology, the patient’s history and clinical signs and laboratory findings helped diagnose the adenoma. The total number of 30 normal pituitary autopsies was collected from cases with no pathological pituitary problems from the Legal Medicine Organization (LMO), Tehran, Iran from January 2017 to June 2020. The anterior epithelial lobe of pituitary with no pathological evidence was collected and normal cases were matched to the patients as a matter of age and gender. The tumor and normal pituitary sample collection and processing was followed as our previous study [24, 25]. The equal number of male and female donors participated in the study with the age range of 44.94 ± 1.17. Notably, both invasive and non-invasive pituitary tumors were collected based on the Knosp classification system where tumor extension to the adjacent sphenoid sinus and cavernous sinus accounts as invasive feature. Also, during the endoscopic surgery, neurosurgeon confirmed tumor invasion. In addition, patients were classified based on the size of their pituitary tumors and the tumor size greater than 10 mm was defined as macro adenomas and the tumor size less than 10 mm was defined as micro adenomas. Following surgical resection, the pituitary fresh tumor tissues were divided into two sections, one transferred to the pathology department for further histological evaluations and the other part was taken away and kept in RNA latter (Qiagen, Germany) immediately and stored in − 80 °C until usage. Notably, we followed the methods of Tavakoli-Yaraki et al. 2019 regarding the pituitary samples collection [24]. The clinic- pathological features of patients with pituitary adenoma are summarized in Table 1.

**Table 1:** The demographic data of the patients with different pituitary adenomas

Variable		Age			Gender			Tumor size		
Patient	Group	≤40	> 40	*P*-Value	Male	Female	*P*-Value	Micro	Macro	*P*-Value
Invasive NFPA (*n* = 18)		4 (22.22%)	14 (77.77%)	0.442	12 (66.66%)	6 (33.33%)	0.1370	0	18 (100%)	
Non-invasive NFPA (*n* = 21)		7 (33.33%)	14 (66.66%)		9 (42.85%)	12 (57.14%)		0	21 (100%)	
Invasive GHPPA (*n* = 14)		6 (42.85%)	8 (57.14%)	0.973	6 (42.85%)	8 (57.14%)	0.8416	3 (21.42%)	11 (78.57%)	0.3854
Non-invasive GHPPA (*n* = 26)		11 (42.30%)	15 (57.69%)		12 (46.15%)	14 (53.14%)		9 (34.61%)	17 (65.38%)	

### RNA extraction, cDNA synthesis, Real-Time PCR

For evaluating the level of necroptosis markers, the harvested tumor and healthy pituitary tissues were subjected to RNA extraction using Trizol (Invitrogen, Grand Island, USA) based on the manufacturer’s instructions. The RNA extraction and Real-Time PCR implementation is followed as the method described by Tavakoli-Yaraki et al. [24]. Briefly, following tissue lysis and homogenize in 700 μL of Trizol lysis reagent, chloroform was applied for phase separation and the upper phase which contain RNA was collected and mixed with isopropanol. Adequate incubation time and centrifugation was performed to obtain RNA pellet which washed with 75% ethanol and air-dried. The quality and quantity of extracted RNA was measured using Nanodrop spectrophotometer (Nanodrop Technologies). The cDNA was synthesized from 1 μg of RNA using PrimeScript First Strand cDNA Synthesis Kit Takara, Japan) according to the manufacturer’s protocol. The synthetized cDNA was applied as a template for Real-Time PCR using SYBR Premix Ex Taq II (Takara, Japan) in an Applied Biosystems Step One Plus, Real time system (Applied Biosystems, USA). The running program was as: 1 cycle at 95 °C for 5 min following 40 cycles at 95 °C for 5 S, 55 °C for 20 S and 60 °C for 35 S. Beta-actin was used as a housekeeping gene with constitutive expression pattern in the pituitary gland to normalize the RIP1K, RIP3K and MLKL gene expression level and the comparative CT (2^-ΔCt^) method was applied for analysis of gene expression. The designed primers which were used are listed in Table 2.

**Table 2:** Primer sets used for qRT-PCR assay

Gene	Primers	Primer sequence	Tm
*RIP1K*	Forward Reverse	5′ –′5′-GCC TTG AAC TTC TGA CCT CA- 3′ 5′- AGC ATC CAC TGA TTG GAC CT − 3′	58
*RIP3K*	Forward Reverse	5’- AGT CTT CCA GAT GGT GGA GA - 3′ 5′- AGC ATC CAC TGA TTG GAC CT - 3′	58
*MLKL*	Forward Reverse	5’- CTG TTA CTT CAG GTT GAG CA - 3′ 5′- GGC AAG GAG ACA GAA CTC AA - 3′	59
*Beta-Actin*	Forward Reverse	5-GAT CTC CTT CTG CAT CCT GT-3′ 5′-TGG GCA TCC ACG AAA CTA C- 3′	57

### Immunohistochemistry

The paraffin-embedded pituitary adenoma tissues were stained with anti-pMLKL monoclonal antibody (Santa Cruz Biotechnology, USA) to assess the protein level of pMLKL via immunohistochemistry. Tissue sections were stained with 1:200 dilutions of anti-pMLKL primary antibody following proper incubation and the anti-mouse IgG HRP-conjugated secondary antibody (Santa Cruz Biotechnology, USA) was applied afterward for 30-min incubation at room temperature. The histological images from tissues were captured by microscope with different magnifications and its in-built digital image software. The semi-quantitative method was applied for slide scoring by independent pathologists and based on the staining intensity and the scores were assigned as negative (absence/weak immunoreactivity) and positive immunoreactivity (immunopositivity). Notably, the positive MLKL staining is localized in the cytoplasm of cells.

### Statistical analysis

The gene expression analysis was calculated using the comparative CT (2^-ΔCt^) method that ΔCt corresponds to the Ct of the target genes (RIP1K, RIP3K and MLKL) subtracted from the Ct of the endogenous gene (Beta-Actin). To determine whether data distributed normally in patients and healthy groups, the Kolmogorov-Smirnov analysis was applied. Also, the chi-square test was used to calculate the statistical differences between patients’ clinic pathological characteristics (age, gender, tumor size) and between-group comparison of enumeration data also the enumeration data are presented as percentages. To analyze the difference between the expression of RIP1K, RIP3K and MLKL between pituitary tumor groups and normal tissues, a t-test and one-way analysis of variance (ANOVA) were applied. The correlation between RIP1K, RIP3K and MLKL gene expressions with patient’s clinic pathological features was analyzed separately for each gene using Pearson coefficient test. Also, the correlation between MLKL gene expression with MLKL protein expression was analyzed using Pearson coefficient test in patients with NFPA and GHPPA. The intra-correlation and inter-correlation of RIP1K, RIP3K and MLKL gene expression was analyzed using Pearson coefficient test. The Graph Pad Prism version 6 (Graph Pad Software, San Diego California) and Statistical Package for Social Science (SPSS v.20) were used for calculation of all statistics. Data were presented as mean ± SD and differences were taken significant for *P* < 0.05, *P* < 0.01 and *P* < 0.001 which were determined by asterisk in related figures.

## Results

### The expression level of RIP1K in NFPA and GHPPA tumor tissues

**Fig. 1:**
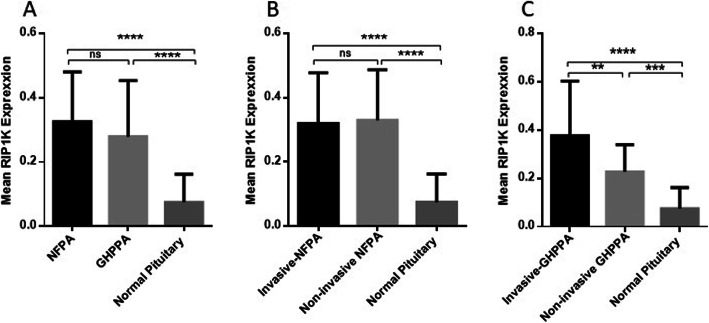
RIP1K expression level in tumor and normal tissues of pituitary. The expression level of RIP1K was evaluated in tumor tissues of NFPA and GHPPA and normal pituitary tissues. The RIP1K expression level was increased in tumor tissues of pituitary adenoma (**A**), invasive and non-invasive NFPA adenoma (**B**), invasive and non-invasive GHPPA (**C**) compared to normal pituitary tissues The Statistical differences between groups were analysed and denoted by asterisk (** = P < 0.01, *** = P < 0.001, **** = P < 0.0001)

The RIP1K gene expression level was evaluated using Real-Time PCR in 109 pituitary tissues including 79 pituitary adenoma tumors and 30 normal pituitary tissues. As shown in Fig. 1A, the expression level of RIP1K revealed a significant increase in NFPA and GHPPA tumors comparing to normal tissues (*P* < 0.0001). The mean and Std. Deviation of RIP1K mRNA level which was calculated using 2^-ΔCt^ method, was 0.3247 ± 0.1556, 0.2790 ± 0.1742, 0.07437 ± 0.08731in NFPA, GHPPA and normal pituitary tissues, respectively. However, no specific difference was observed regarding the expression level of RIP1K between NFPA and GHPPA tumors. Moreover, comparing the expression level of RIP1K between invasive (0.3195 ± 0.1576) and non-invasive (0.3291 ± 0.1577) NFPA showed no statistically difference; while remarkable elevation of RIP1K expression was detected in invasive and non-invasive NFPA compared to normal pituitary tissues (*P* < 0.0001) (Fig. 1B). In addition, a significant elevation in the level of RIP1K was observed in GHPPA invasive (*P* < 0.0001) and non-invasive tumors (*P* < 0.001) compared to normal pituitary tissues (Fig. 1C). The RIP1K mRNA level was 0.3754 ± 0.2271 and 0.2270 ± 0.1118 in invasive and non-invasive GHPPA pituitary tumors which showed significant difference (*P* < 0.01). To better determine how the RIP1K gene expression and patient’s demographic features are related, the correlation of RIP1K expression with the aforementioned features was evaluated for each tumor type separately and the results are presented in Table 3. It was revealed that RIP1K gene expression showed no significant correlation with patient’s age, gender and tumor size. Also, it was revealed that RIP1K gene expression was not significantly correlated with the expression level of RIP3K and MLKL in patients with GHPPA (Table 5) and NFPA (Table 6).

**Table 3:** Correlation of RIP1K expression with clinic pathological features of the patients

Variable	Pearson coefficient test	Invasive GHPPA	Non-invasive GHPPA	Invasive NFPA	Non-invasive NFPA
**Age**	Correlation *P*-value	0.2034 0.518	−0.015 0.94	0.231 0.3544	0.000 > 0.9
**Gender**	Correlation *P*-value	0.166 0.606	0.108 0.599	−0.08 0.75	0.048 0.834
**Tumor size**	Correlation *P*-value	0.064 0.88	0.07 0.732	−0.04 0.87	−0.4 0.08

### The RIP3K expression level in NFPA and GHPPA tumor tissues

**Fig. 2:**
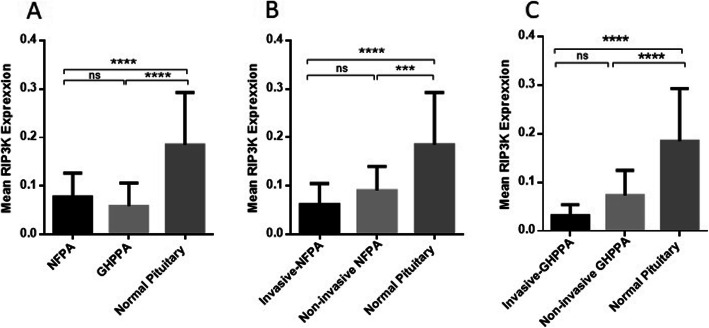
RIP3K expression levels in tumor and normal tissues of pituitary. The expression levels of RIP3K were evaluated in tumor and normal tissues of pituitary with different adenoma types. The RIP3K expression level was increased in tumor tissues of NFPA and GHPPA (**A**) invasive and non-invasive NFPA adenoma (**B**), invasive and non-invasive GHPPA (**C**) compared to normal pituitary tissues. The Statistical differences between groups were analysed and denoted by asterisk (*** = P < 0.001, **** = P < 0.0001)

Since RIP3K is the key factor mediating necroptosis, the expression level of RIP3K was also evaluated in tumor tissues and normal pituitary gland. Based on our results, the expression level of RIP3K was significantly reduced in tumors comparing to normal pituitary tissues (*P* < 0.0001) (Fig. 2A). The mean and Std. Deviation of RIP3K mRNA levels were 0.07797 ± 0.04855, 0.0586 ± 0.04755 and 0.1849 ± 0.1080 in NFPA, GHPPA and normal pituitary tissues, respectively (Fig. 2A). Based on the results no significant difference was detected between the expression level of RIP3K in NFPA and GHPPA tumors. In accordance, invasive (*P* < 0.0001) and non-invasive (*P* < 0.001) NFPA tumors expressed lower level of RIP3K compared to normal pituitary tissues (Fig. 2B); while the RIP3K expression level showed no statistically difference between invasive (0.06159 ± 0.04308) and non-invasive (0.09064 ± 0.04965) NFPA tumors. The same pattern was observed regarding the level of RIP3K in GHPPA subtypes; since invasive (0.03129 ± 0.02241) and non-invasive (0.07331 ± 0.05122) GHPPA tumors expressed significantly lower level of RIP3K compared to normal pituitary tissues (*P* < 0.0001); while the difference between them was not remarkable (Fig. 2C). The correlations between RIP3K expression levels and patient’s clinic pathological features are summarized in Table 4 which delineate significant correlation of non-invasive GHPPA (*P* = 0.03) with patient’s age.; while no significant correlation was detected regarding the expression level of RIP3K and tumor size, gender and age in NFPA and GHPPA group. Notably, it was shown that RIP3K expression level was significantly correlated with the expression level of MLKL in patients with GHPPA (Table 5); while the correlation between RIP3K and MLKL in NFPA group was not remarkable (Table 6).

**Table 4:** Correlation of RIP3K expression with clinic pathological features of the patients

Variable	Pearson coefficient test	Invasive GHPPA	Non-invasive GHPPA	Invasive NFPA	Non-invasive NFPA
**Age**	Correlation *P*-value	−0.074 0.822	0.411 0.03	0.232 0.354	0.05 0.82
**Gender**	Correlation *P*-value	−0.26 0.381	0.072 0.726	0.17 0.49	0.081 0.72
**Tumor size**	Correlation *P*-value	0.215 0.458	−0.123 0.547	−0.119 0.635	− 0.16 0.47

**Table 5:** Intra-correlation of necroptosis pathway mediators in GHPPA

	Pearson coefficient	RIP1K	RIP3K	MLKL
**RIP1K**	Correlation	1.000	0.17	0.21
	*p*-value		0.401	0.401
**RIP3K**	Correlation	0.17	1.000	0.62
	*p*-value	0.401		0.00044
**MLKL**	Correlation	0.21	0.62	1.000
	*p*-value	0.401	0.0004

**Table 6:** Intra-correlation of necroptosis pathway mediators in NFPA

	Pearson coefficient	RIP1K	RIP3K	MLKL
**RIP1K**	Correlation	1.000	0.123	−0.05
	*p*-value		0.6	0.825
**RIP3K**	Correlation	0.123	1.000	0.38
	*p*-value	0.6		0.092
**MLKL**	Correlation	−0. 05	0.38	1.000
	*p*-value	0.825	0.092	

### The MLKL expression level in NFPA and GHPPA tumor tissues

**Fig. 3:**
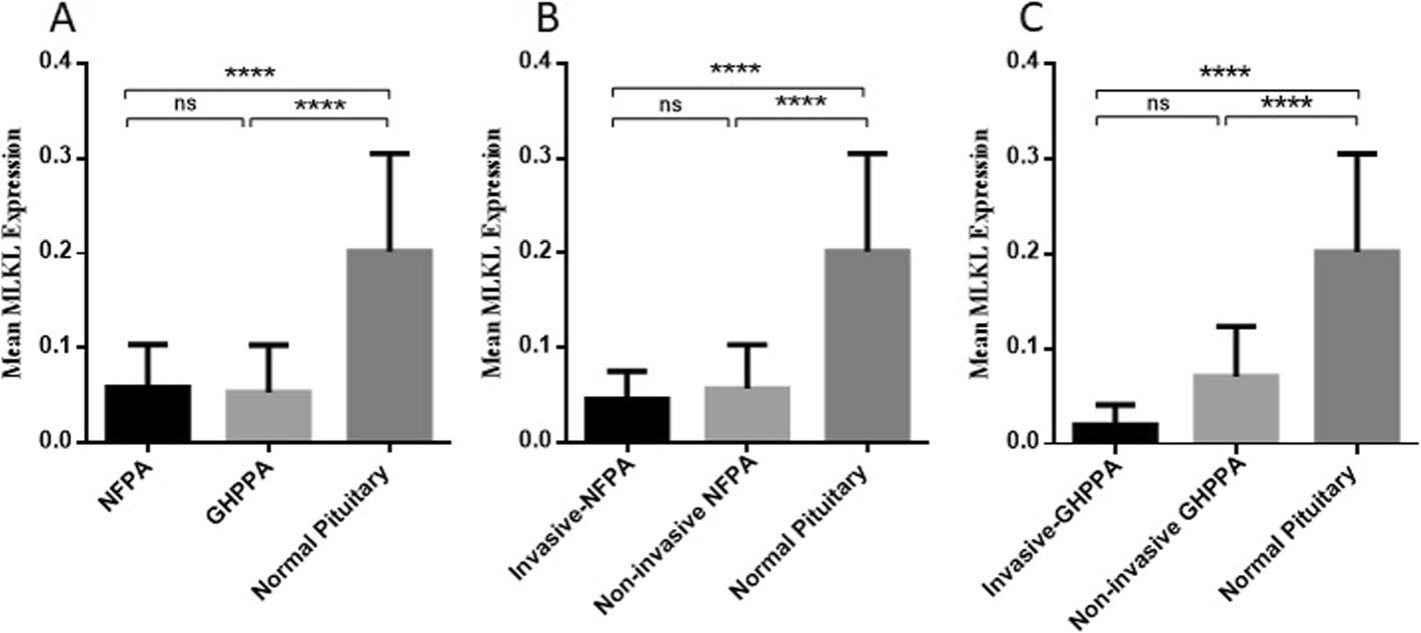
MLKL expression levels in tumor tissues of pituitary adenoma. The expression level of MLKL was evaluated in tumor tissues of NFPA and GHPPA and normal pituitary tissues. The MLKL expression level was increased in tumor tissues of pituitary adenoma (**A**), invasive and non-invasive NFPA adenoma (**B**), invasive and non-invasive GHPPA (**C**) compared to normal pituitary tissues The Statistical differences between groups were analysed and denoted by asterisk (**** = P < 0.0001)

To get insight into the execution of necroptosis, the expression level of MLKL was also investigated in different pituitary adenoma and normal tissues. As it is demonstrated in Fig. 3A, the expression level of MLKL was significantly decreased in NFPA and GHPPA adenoma comparing to normal tissues (*P* < 0.0001). The mean and Std. Deviation of MLKL mRNA level was 0.05811 ± 0.04531, 0.052750.05034 and 0.2018 ± 0.1033 in NFPA, GHPPA and normal pituitary tissues, respectively (Fig. 3A). In comparing the level of RIP3K expression in NFPA subtypes, it was revealed that the MLKL expression level was significantly decreased in invasive (0.04508 ± 0.02991) and non-invasive (0.05657 ± 0.04615) NFPA tumors compared to normal pituitary tissues (*P* < 0.0001); while no specific difference was observed between NFPA tumors (Fig. 3B). In accordance, invasive and non-invasive GHPPA showed lower expression level of MLKL compared to normal pituitary tissues (*P* < 0.0001) (Fig. 3C). Despite of lower MLKL expression level in invasive GHPPA (0.01969 ± 0.02137), no significant difference was observed compared to non-invasive GHPPA tumors (0.07056 ± 0.05267). The correlations between MLKL gene expression level and patient’s clinic pathological features which is summarized in Table 7 showed that MLKL expression was significantly correlated with tumor size in invasive NFPA group (*P* = 0.039); while no other remarkable correlation was detected regarding the MLKL expression with tumor size, age and patient’s gender in GHPPA and NFPA group.

**Table 7:** Correlation of MLKL expression with clinic pathological features of the patients

Variable	Pearson coefficient test	Invasive GHPPA	Non-invasive GHPPA	Invasive NFPA	Non-invasive NFPA
**Age**	Correlation *P*-value	−0.166 0.582	0.082 0.689	0.437 0.069	−0.025 0.91
**Gender**	Correlation *P*-value	−0.18 0.54	0.05 0.78	0.272 0.273	0.162 0.48
**Tumor size**	Correlation *P*-value	0.108 0.747	0.084 0.682	0.463 0.039	−0.354 0.149

### The reduced expression of MLKL protein in pituitary adenoma tumor tissues

**Fig. 4:**
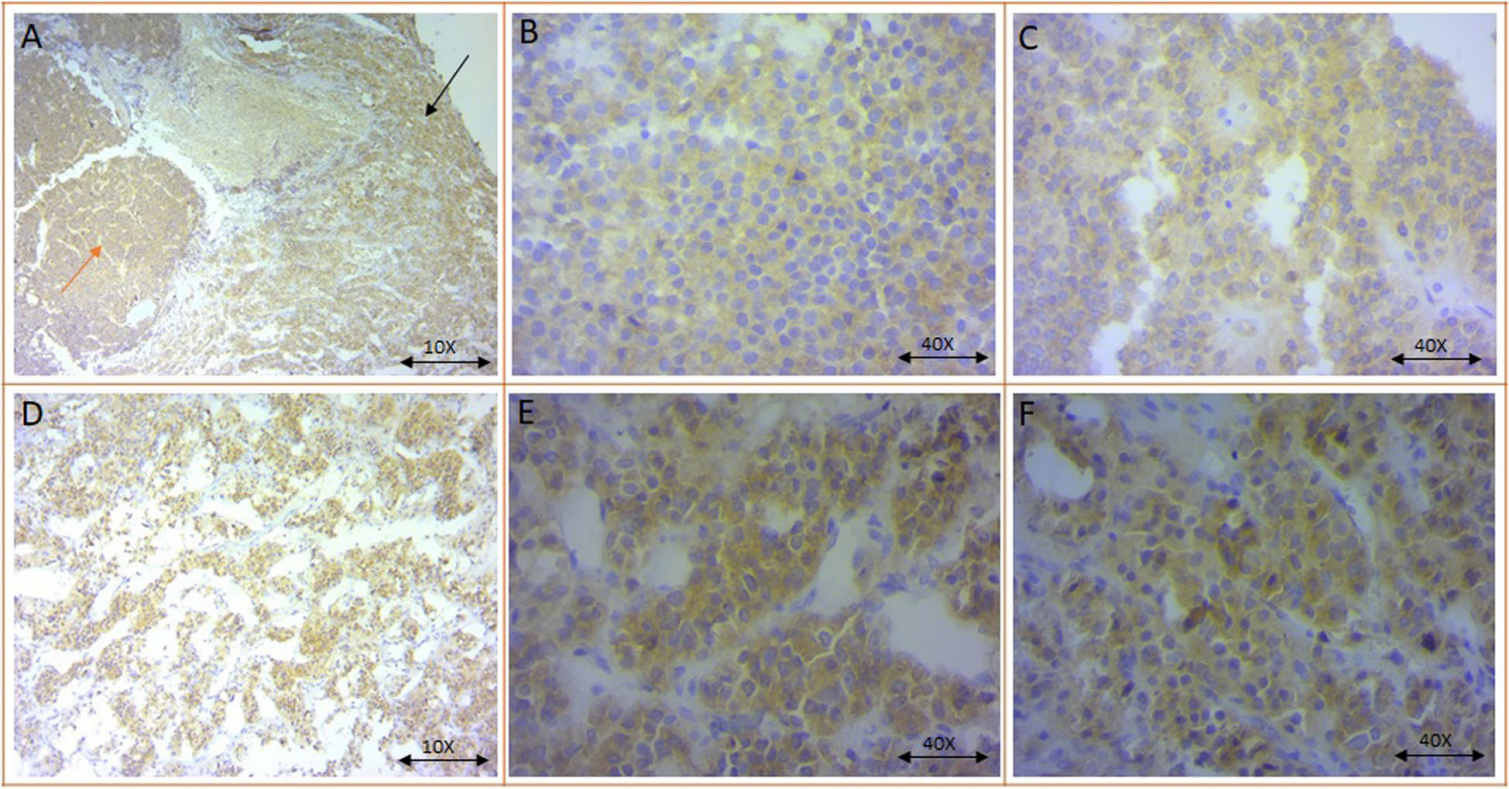
The representative images of MLKL immunohistochemistry staining in pituitary adenoma and normal tissues. Differential protein expression of MLKL was evaluated by immunohistochemistry in NFPA and GHPPA and normal pituitary tissues. **A** Indicates hot-spot regions for tumor (red arrow) and normal (black arrow) sections, × 10., **B** Indicates no immunopositivity in NFPA tumor tissue, × 40., **C** Indicates no immunopositivity in GHPPA tumor tissue, × 40., **D** Indicates normal pituitary tissue, positive immunoreactivity, × 10., **E**, **F** Indicates cytoplasmic immunopositivity in normal pituitary tissue, × 40

According to delineate the MLKL expression pattern in pituitary adenoma tissues, a question might be raised that whether the MLKL-reduced mRNA level is accompanied with low MLKL protein level in pituitary adenomas. To address this, the protein level of pMLKL was evaluated in NFPA and GHPPA adenoma and normal tissues using immunohistochemistry. Moreover, the representative images of MLKL immunohistochemistry staining in pituitary tumor and normal tissues are illustrated in Fig. 4. Based on our data that is summarized in Table 8, it was revealed that 69.23 and 62.5% of NFPA and GHPPA tumors was negative for MLKL protein level. Also, majority of invasive and non-invasive NFPA tumors showed no expression for MLKL protein; since 22.22 and 38.01% of invasive and non-invasive NFPA tumors positively expressed MLKL, respectively. In addition, 71.42 and 57.69% of invasive and non-invasive GHPPA tumors were negative for MLKL protein expression, respectively. Regarding GHPPA tumors as a matter of tumor size, it was observed that 81.81% of invasive macro adenoma GHPPA were negative for MLKL expression; while 66.66% of invasive GHPPA with micro adenoma were positive for MLKL protein. In non-invasive GHPPA tumors regarding tumor size the difference was not remarkable and 55.55 and 58.82% of non-invasive micro- and macro- GHPPA tumors negatively expressed MLKL protein. Although it seems that invasive NFPA and GHPPA expressed lower level of MLKL protein compared to non-invasive tumors in each group, the differences were not enough to be statistically significant. Notably, to evaluate the possible relevance of MLKL gene expression with MLKL protein expression level in tumor tissues, a correlation was evaluated between MLKL gene and protein expression in NFPA and GHPPA groups that are demonstrated in Table 9. Based on our data, MLKL gene expression in patients with large tumor size (macro adenoma) was significantly correlated with MLKL protein level in NFPA (*P* = 0.0091) and GHPPA (*P* = 0.0149) tumors. Also, the MLKL gene expression level in invasive pituitary tumors was correlated significantly with MLKL protein level in NFPA (*P* = 0.0243) and GHPPA (*P* = 0.0308) tumors.

**Table 8:** The immunohistochemistry results of MLKL protein evaluation in patients with pituitary adenoma

Group	Type	Total number	MLKL expression Negative		MLKL expression Positive		*P* value
			%	N	%	N	
Adenoma type	NFPA	39	27	69.23%	12	30.76%	0.5283
	GHPPA	40	25	62.5%	15	37.5%	
NFPA	Invasive	18	14	77.77%	4	22.22%	0.2843
	Non-invasive	21	13	61.9%	8	38.01%	
GHPPA	Invasive	14	10	71.42%	4	28.57%	0.3920
	Non-invasive	26	15	57.69%	11	42.30%	
	Invasive Micro-adenoma	3	1	33.33%	2	66.66%	0.0994
	Invasive Macro-adenoma	11	9	81.81%	2	18.18%	
	Non-invasive Micro-adenoma	9	5	55.55%	4	44.44%	0.8725
	Non-invasive Macro-adenoma	17	10	58.82%	7	41.17%

**Table 9:** The MLKL gene expression correlation with MLKL protein level in patients with pituitary adenoma

Variable	Pearson coefficient test	MLKL protein expression in GHPPA	MLKL protein expression in NFPA
**Tumor MLKL gene expression**	Correlation *P*-value	0.3170 0.0463	0.3386 0.0350
**Macro adenoma MLKL gene expression**	Correlation *P*-value	0.3873 0.0149	0.4925 0.0091
**Micro adenoma MLKL gene expression**	Correlation *P*-value	0.05391 0.8428	
**Invasive tumor MLKL gene expression**	Correlation *P*-value	0.5983 0.0308	0.4785 0.0243
**Non-invasive tumor MLKL gene expression**	Correlation *P*-value	0.05150 0.8027	0.3894 0.1224

## Discussion

It is speculated that the transformed cells undergo genetic defects or mutations which should be removed via cell death pathways before initiate tumor formation [11, 26]. The defective cell death lead to switch of cell proliferation/death balance in favor of cell growth and non-efficient tumor cell elimination [7]. Rationally, in the pituitary adenomas, the cell death pathways should be attenuated which culminate in cell growth, however few studies are focused on characterizing the possible cell death pathways in pituitary adenomas [27]. More recently, it was shown that bromocriptine as a dopamine (DA) receptor agonists which used as a common therapeutic option for prolactinoma can induce RIP3K and pMLKL-positive cells and protein level in prolactinoma patients under bromocriptine therapy. Additionally, bromocriptine treatment induced the cell viability and ATP decrease also ROS level increase in prolactinoma cell line which were diminished following necroptosis specific administration. However, the status of necroptosis biomarker expression pattern in prevalent pituitary adenomas is lacking which is perused in the current study. Based on the result of the current study, RIP1K over-expressed significantly in NFPA and GHPPA tumor tissues compared to normal pituitary tissues. Also, the elevated expression level of RIP1K was detected in invasive GHPPA compared to non-invasive GHPPA tumors; while no difference in the level of RIP1K was observed between invasive and non-invasive NFPA tumors. Ize. The elevated expression level of RIP**1**K was also observed in non-small cell lung cancer which promotes tumor cell transformation via ROS overproduction [28]. Similarly, it was shown that RIP1K silencing resulted in suppressing melanoma cell proliferation and the oncogenic effect of RIP1K was mediated through activation of NF-κB pathway in melanoma [29]. In support of this, the elevated level of RIP1K caused suppressing induction of DNA damage and triggering p53 which was implemented via NF-κB pathway [30]. Also, it was reported that the expression level of RIP1K was elevated in breast cancer tumors with different tumor severity resulting in poor prognostic value [16]. Multiple evidences revealed that RIP1K emerged as a dual function enzyme regarding malignant cell proliferation with kinase-dependent and kinase-independent activity. The positive effects of RIP1K in the regulation of NF-κB pathway enable the cells to express pro-survival mediators. On the other hand, it was observed that the possibility of RIP1K ubiquitination following TNF activation lead to prevention of cell death complex formation [31]. Consequently, the absence of RIP1K provides the situation for RIP3K and MLKL activation and necroptosome complex formation [14]. According to our results, invasive pituitary adenomas exhibited higher level of RIP1K expression which is in line with the oncogenic role of RIP1K and its increased expression in tumors with higher grades [16]. Our results showed considerable decrease in the expression level of RIP3K in the tumor tissues of pituitary adenomas. In agreement with our results, the lower expression level of RIP3K was observed in malignant breast tumors and also MDA-MB-468 cells which were restored in response to shikonin treatment via overproduction of ROS and reduction of mitochondrial membrane potential [16, 17]. Also, the silenced expression of RIP3K in breast cancer might be due to the methylation of RIP3K transcription site that can be considered in chemotherapy-induced cell death approaches [32]. The reduced expression level of RIP3K was also observed more in invasive versus non-invasive NFPA and GHPPA tumors; however, no significant correlation was obtained regarding tumor size with RIP3K expression both groups. The RIP3K gene expression was highly correlated with.

MLKL gene expression in GHPPA group; while the correlation of RIP3K and MLKL was not statistically significant in NFPA group.. Based on our data, the expression level of MLKL was significantly decreased in pituitary tumors which was associated with reduced level of pMLKL protein in pituitary adenoma tissues. The lower expression of MLKL was also detected in invasive versus non-invasive, macro adenome versus micro adenoma of NFPA and GHPPA tumors. Moreover, the pMLKL negative protein level was observed in 69.23% of NFPA tumors while 62.5% of GHPPA revealed negative level of pMLKL level; however, the difference was not significant. In fact, NFPAs normally have larger tumor size which might reflect the reduced cell death rate and accelerated cell proliferation rate and apoptosis since it was shown that the apoptotic index was remarkably lower in NFPAs comparing to functional pituitary adenomas. The higher rate of cell proliferation in NFPAs might explain the lower expression of MLKL gene and its protein level in tumor tissues of patients with NFPAs in the current study. It is well-documented that the MLKL expression, as a key downstream mediator and executor of necroptosis, is under regulation of RIP3K [33]. The similar expression pattern of RIP3K-MLKL in pituitary adenoma tissues might provide more data on the possible involvement of necroptosis death pathway in the pituitary cell growth however further mechanistic studies are required to unravel the exact role of RIP1K, RIP3K and MLKL in pituitary tumor cell fate. Although our study provides first evidences regarding the expression profile of necroptosis main biomarkers in pituitary adenomas, the necroptosis is a complex death pathway which is dependent to several mediators as well as cell physiology and environment therefore characterizing the exact role of necroptosis pathway in pituitary pathogenesis should be clarified in broader mechanistic studies. Notably, necroptosis is a kinase-dependant death pathways that might be triggered by tumor necrosis factor receptor 1 (TNFR1) death receptor, Toll-like receptors (TLRs) or intracellular sensors. Dependent to the type of receptor which activate necroptosis, different cellular mediators might be involved. Following TNFR1 activation, RIP1K is recruited which leads to RIP3K phosphorylation, oligomerization and MLKL engagement. However, the TLRs activation induce RIP3K activation in a RIP1K-independent manner although the exact mechanism has yet to be defined. In accordance, the inconsistency regarding the expression pattern of RIP1K and RIP3K in our pituitary adenoma tissues might suggest that TLRs or intracellular sensors are responsible for triggering necroptosis in pituitary adenoma cells which should be proved by further studies.. To achieve more robust results, it is suggested to study the mediators of necroptosis pathway in more patients with NFPA and GHPPA tumors as well as patients with other types of pituitary tumors. Also, the study of RIP1K and RIP3K protein levels along with the study of the above genes can be helpful to reach a solid conclusion. Moreover, based on the fact that the caspase-8 inhibition is prerequisite for necroptosis activation, simultaneous evaluation of caspase-8 beside necroptosis biomarkers can yield promising data.

## Conclusions

Due to the crucial role of programmed necrosis in the regulation of cancer cell growth, as well as little knowledge regarding the cellular signaling pathways responsible for pituitary tumor formation and progression, the results of the current study may open up new insights toward the possible impact of necroptosis in pathogenesis of pituitary adenomas and indicates that necroptosis pathway attenuation might lead pituitary cells to proliferation and tumor formation, although this requires mechanistic evaluation.

## Data Availability

All data generated or analyzed during this study and supporting our findings are included and can be found in the manuscript. The raw data can be provided by the corresponding author on reasonable request.

## Abbreviations

RIPKs: Receptor-interacting protein kinases
NF-κB: Nuclear factor kappa-light-chain-enhancer of activated B cells
MAP kinase: Mitogen-activated protein kinase
FPA: Functional pituitary adenoma
NFPA: Non-functional pituitary adenomas
ETSS: Endoscopic transnasal transphenoidal surgery
HPA: Hypothalamic-pituitary-adrenal
ROS: Reactive oxygen species
STAT: Signal transducer and activator of transcription
TNF: Tumor necrosis factor
TRADD: TNFRSF1A-associated death domain
FADD: Fas-associated death domain
MLKL: Mixed lineage kinase domain-like
